# Dynamic emotion recognition and learning motivation prediction in Chinese second language acquisition under cultural differences: a study based on ED-CM-MP model

**DOI:** 10.3389/fpsyg.2025.1743759

**Published:** 2026-01-21

**Authors:** Jing Hao

**Affiliations:** College of Literature and Journalism, Yan'an University, Yan'An, Shaanxi, China

**Keywords:** Chinese L2 acquisition, cultural differences, DistilBERT, dynamic emotion, GraphSAGE, learning motivation prediction

## Abstract

To address the core issues of low accuracy, poor cultural adaptation, and insufficient efficiency in learning motivation prediction in cross-cultural Chinese second language acquisition scenarios, this paper proposes the ED-CM-MP model, which integrates dynamic sentiment recognition, cultural adjustment modeling, and lightweight temporal prediction. This model uses DistilBERT+Gated TCN to construct a dynamic sentiment module to extract temporal sentiment features, GraphSAGE to adjust for cross-cultural differences, and Temporal Fusion Transformer to achieve efficient motivation prediction. Experiments on the HSK and VIDAS cross-cultural datasets show that the model achieves the best core prediction performance: MAE of 0.28–0.29 and F1 Score of 0.91–0.92, representing a 10.2% improvement in accuracy compared to the best baseline model; inference latency as low as 38.5–39.2 ms and FLOPs of only 12.6–13.1 G, representing a 20.3% improvement in efficiency compared to MobileNetV3; and a cultural adaptation score of 0.94–0.95, representing a 21.8% improvement in cross-cultural generalization ability compared to U-Cast. Ablation experiments validated the necessity of the three modules working together; removing any module resulted in a performance decrease of 3.6%-7.2%. Stability tests showed that the model exhibited excellent robustness with performance fluctuations of ≤ 5.4% in a small sample scenario with 10% labeled noise and 2000 training samples. This research demonstrates that the ED-CM-MP model achieves a triple breakthrough in motivation prediction–accuracy, efficiency, and generalization–providing an efficient and feasible technical solution for intelligent teaching intervention in cross-cultural Chinese second language acquisition.

## 1 Introduction

With the acceleration of globalization and the rapid development of Chinese international education, Chinese as a Second Language (Chinese L2) acquisition has become a hot research topic in the field of cross-cultural communication. Accurately capturing learners' emotional states and learning motivation is of significant practical importance for optimizing teaching strategies and improving acquisition outcomes (Yu and Liu, 2024). Emotion, as a core psychological variable in the second language acquisition process, directly influences learners' cognitive engagement and learning persistence through its dynamic fluctuations (such as the phase changes of learning anxiety or the real-time fluctuations of learning interest) (Ding and Xing, 2022). Learning motivation, as the core driving force behind learning behavior, is even more crucial, with its intensity and changing trends determining the effectiveness of language acquisition. Importantly, under cross-cultural contexts, significant differences exist between cultural groups (e.g., East Asian learners with a collectivist orientation and Western learners with an individualistic orientation) in terms of emotional expression, learning cognition, and motivation formation mechanisms (Bovolenta and Marsden, 2022). The moderating role of cultural factors on emotional dynamics and learning motivation is becoming increasingly prominent, which also raises higher demands for dynamic modeling techniques in intelligent educational scenarios.

Although existing research has begun to focus on emotion recognition and motivation prediction in second language acquisition, three core research gaps remain to be addressed. First, in emotional modeling, most studies still rely on single-time static measurements or single-modal analysis, depending on traditional methods such as questionnaires and classroom observations (Karimi et al., 2022). These approaches struggle to capture the dynamic temporal features of emotions as they change across learning phases and task types, and lack the integration and exploration of multimodal data (such as text interactions and learning behaviors), limiting the real-time accuracy of emotion recognition. Second, in the depiction of cultural modulation mechanisms, existing models often treat cultural factors as static variables, lacking dynamic modeling of the complex interactions between “cultural dimensions–emotional dynamics–motivation changes” (Li, 2025). These models fail to quantify the differential modulation weights of emotional fluctuations by different cultural backgrounds and are unable to cater to the personalized needs of cross-cultural learners. Finally, in model design, existing methods often struggle to balance the precision of dynamic temporal modeling, the flexibility of cultural adaptation, and the lightweight requirements of deployment applications (Yu et al., 2022). Some models suffer from low inference efficiency due to excessive complexity, while others simplify the design, neglecting critical interaction mechanisms, making them unsuitable for practical teaching applications.

To address the aforementioned research gaps, this paper proposes an integrated deep learning model (ED-CM-MP Model) that combines dynamic emotion recognition, cultural modulation modeling, and learning motivation prediction. This model aims to accurately capture emotional dynamics, effectively characterize cultural modulation mechanisms, and scientifically predict learning motivation in cross-cultural Chinese second language acquisition scenarios. The subsequent content of this paper is arranged as follows: The related research section will systematically review the research progress in dynamic emotion recognition, cultural modulation modeling, and learning motivation prediction in the field of second language acquisition, clarifying the research positioning and core contributions of this paper; the materials and methods section will elaborate on the overall framework, core module design, experimental dataset, baseline model selection, and experimental setup of the ED-CM-MP model; the results and analysis section will verify the model's performance advantages through comparative experiments and ablation experiments, and deeply analyze the cultural modulation mechanisms and model stability; finally, the conclusion section will summarize the research results, objectively analyze the research limitations, and look forward to future research directions.

## 2 Related work

Dynamic emotion recognition in second language (L2) acquisition is a key technology for optimizing teaching interventions and enhancing learning outcomes. The research in this area has evolved from traditional machine learning methods to deep learning-driven temporal modeling, but still faces core issues such as insufficient dynamic feature capture, inaccurate multimodal fusion, and lack of lightweight adaptation (Wang et al., 2024). Early studies often relied on traditional algorithms such as Support Vector Machines (SVM) and Random Forests, using manually extracted emotional words from text, facial expression features, or learning behavior indicators for emotion classification (Adeel and Tao, 2024). Although these methods achieved basic emotion state recognition, they were limited by the subjectivity and constraints of manually extracted features, making it difficult to capture the dynamic fluctuations of emotions such as anxiety and interest, which change with learning stages and task difficulty. Moreover, these methods were not well-suited for the emotional expression differences among cross-cultural learners. With the development of deep learning technologies, Recurrent Neural Networks (RNNs), such as Long Short-Term Memory (LSTM) and Gated Recurrent Units (GRU), have been widely applied to dynamic emotion recognition due to their ability to model temporal sequences (Zheng, 2022). By extracting emotional patterns from sequential data, such as learning logs and classroom interaction texts, these models significantly improved recognition accuracy. However, these models often encounter the gradient vanishing problem when processing long temporal sequences, making it difficult to effectively capture long-term emotional dependencies. In recent years, pre-trained language models based on Transformer architecture, such as BERT and RoBERTa, have shown superior performance in text-based emotion recognition tasks due to their powerful semantic understanding capabilities (Zhang et al., 2023). Some studies have further combined these models with temporal models to enhance dynamic feature extraction. However, most models still have two significant shortcomings: first, they focus primarily on the single text modality, with crude fusion strategies for multimodal data such as speech tone and learning behavior, failing to fully leverage complementary multidimensional information to improve dynamic recognition (Tian and McCafferty, 2022); second, the models are complex with large parameter sizes, resulting in lower inference efficiency, which makes them difficult to adapt to the practical needs of cross-device deployment and real-time teaching interventions in Chinese L2 acquisition scenarios. Additionally, these models lack sufficient adaptability to the unique emotional expression habits of Chinese language learners, such as implicit emotional expressions in text and task-driven emotional fluctuations. These limitations provide the core direction for improvement in the construction of the “DistilBERT + Gated TCN” lightweight dynamic emotion recognition module in this paper.

In cross-cultural learning scenarios, cultural modulation modeling, as a core research direction to reveal the mechanisms of cultural differences in second language (L2) acquisition, has always focused on the question of “how to precisely capture the dynamic interaction between cultural factors and the learning process.” However, there are still prominent issues such as the simplistic representation of cultural dimensions, static processing models, and insufficient cross-cultural adaptability (Fernandes et al., 2024). Early research mainly relied on Hofstede's cultural dimension theory (e.g., collectivism/individualism, power distance) or Schumann's second language acquisition cultural adaptation framework, using statistical analysis methods to explore the correlation between cultural background and learning outcomes (Pasquarella et al., 2025). Although the significant moderating effect of cultural factors was confirmed, culture was often treated as a static categorical variable, with cultural association analysis achieved only through group comparisons or simple feature embeddings. This approach fails to capture the real-time dynamic moderation effect of culture on emotional fluctuations and motivation changes during the learning process and overlooks specific cultural adaptation issues in the Chinese L2 acquisition context, such as the cognitive differences between learners from Chinese-character cultures and non-Chinese-character cultures. With the development of natural language processing technology, multilingual pre-trained models (e.g., Multilingual-BERT, XLM-RoBERTa) have become mainstream tools for cultural modulation modeling (Meunier et al., 2022). These models enhance the adaptability to language expressions from different cultural backgrounds by pre-training on multilingual corpora. Some studies have further incorporated cultural labels to improve the cultural sensitivity of the models, but there are still obvious flaws: on one hand, these models focus primarily on cultural adaptation at the language level, lacking modeling of the complex interaction mechanisms between “cultural dimensions–emotional dynamics–motivation formation,” and they struggle to quantify the differential modulation weights of various cultural factors during the learning process (Cui et al., 2025). On the other hand, the cultural modulation logic of existing models is often based on generic cross-linguistic scenario designs, without fully considering the unique cultural cognitive characteristics of Chinese L2 learners (e.g., learners from collectivist cultures being more influenced by peers' emotions) (Acs et al., 2024), which limits the modulation effect in Chinese cross-cultural learning scenarios. Although recent studies have attempted to construct cultural-learner association graphs using graph neural networks, these efforts have remained at the level of simple node connections and failed to deeply integrate cultural features with dynamic learning features such as emotion and behavior. These research gaps provide the core entry point for this paper's introduction of the GraphSAGE graph neural network to construct a heterogeneous graph structure, enabling the dynamic and refined modeling of cultural modulation, as well as offering new technical approaches to address cultural adaptation challenges in Chinese cross-cultural second language acquisition.

Learning motivation prediction for second language (L2) learners is a key support for personalized teaching interventions and enhancing learning persistence. The research in this area has gradually shifted from traditional statistical analysis to data-driven temporal prediction. However, current studies still face core issues such as insufficient dynamic driving mechanism modeling, inadequate multi-factor integration, and lack of cross-cultural adaptability (Yang and Wu, 2022). Early research mainly employed traditional statistical methods like regression analysis and structural equation modeling to build static association models between “learning attitude–learning engagement–motivation level” for motivation prediction. Although these studies identified some influencing factors (e.g., learning goals, teacher feedback), they struggled to capture the dynamic characteristics of motivation changes due to learning progress, emotional fluctuations, and cultural adaptation processes (Van Mensel and Galand, 2023). Additionally, most prediction results are limited to a unidimensional assessment of motivation strength, lacking accurate forecasts of future motivational trends. With the application of machine learning techniques, models such as Random Forest and Support Vector Regression have improved prediction accuracy by integrating learning behavior data (e.g., learning duration, practice accuracy). However, these models still rely on handcrafted feature engineering, with insufficient exploration of latent driving factors such as emotional states, and fail to effectively utilize dynamic relational information in temporal data, which limits prediction timeliness and generalization ability (Wu, 2022). In recent years, temporal prediction models (e.g., Temporal Fusion Transformer, PatchTST) have become research hotspots due to their powerful ability to capture dynamic features (Xie et al., 2025). Some studies have attempted to integrate emotional features to improve prediction outcomes, but there are still significant flaws: first, emotion is often treated as a static input variable, ignoring the real-time driving effect of dynamic emotional fluctuations on motivation changes, and failing to build a closed-loop connection between “emotional dynamics–motivation prediction” (Kusumawardani and Alfarozi, 2023); second, cultural factors are not adequately considered, and the motivational differences in cross-cultural learners (e.g., the weight of instrumental motivation in different cultural groups) are not fully integrated, resulting in poor adaptability in cross-cultural Chinese L2 acquisition scenarios; third, multi-feature fusion strategies are relatively simple, and deep interactive modeling of dynamic emotions, static culture, and learning behavior features has not been achieved, affecting prediction accuracy and interpretability. These research gaps not only limit the practical application of motivation prediction models in Chinese L2 acquisition but also provide the core.

Based on the progress and shortcomings of the aforementioned related research, the core contributions of this paper are mainly reflected in three aspects:

1. A lightweight dynamic sentiment recognition module based on “DistilBERT+Gated TCN” was constructed, which efficiently captures long-term temporal dependency features of sentiment while ensuring semantic understanding accuracy, solving the problems of insufficient dynamic modeling and excessive computational consumption of traditional models;
2. GraphSAGE graph neural network was introduced to realize dynamic modeling of cultural modulation. By constructing a heterogeneous graph structure of “learner-cultural dimension-sentiment type”, the contribution of different cultural factors to the dynamic modulation of sentiment was quantified, filling the gap in existing research on the insufficient characterization of the culture-sentiment interaction mechanism;
3. A motivation prediction module based on temporal fusion Transformer (TFT) was designed to effectively integrate dynamic sentiment features, static cultural features, and learning behavior features, improving the accuracy and interpretability of motivation prediction and providing new technical support for the intelligent upgrading of cross-cultural Chinese as a second language teaching.

## 3 Methods and materials

### 3.1 ED-CM-MP model

**Figure 1:**
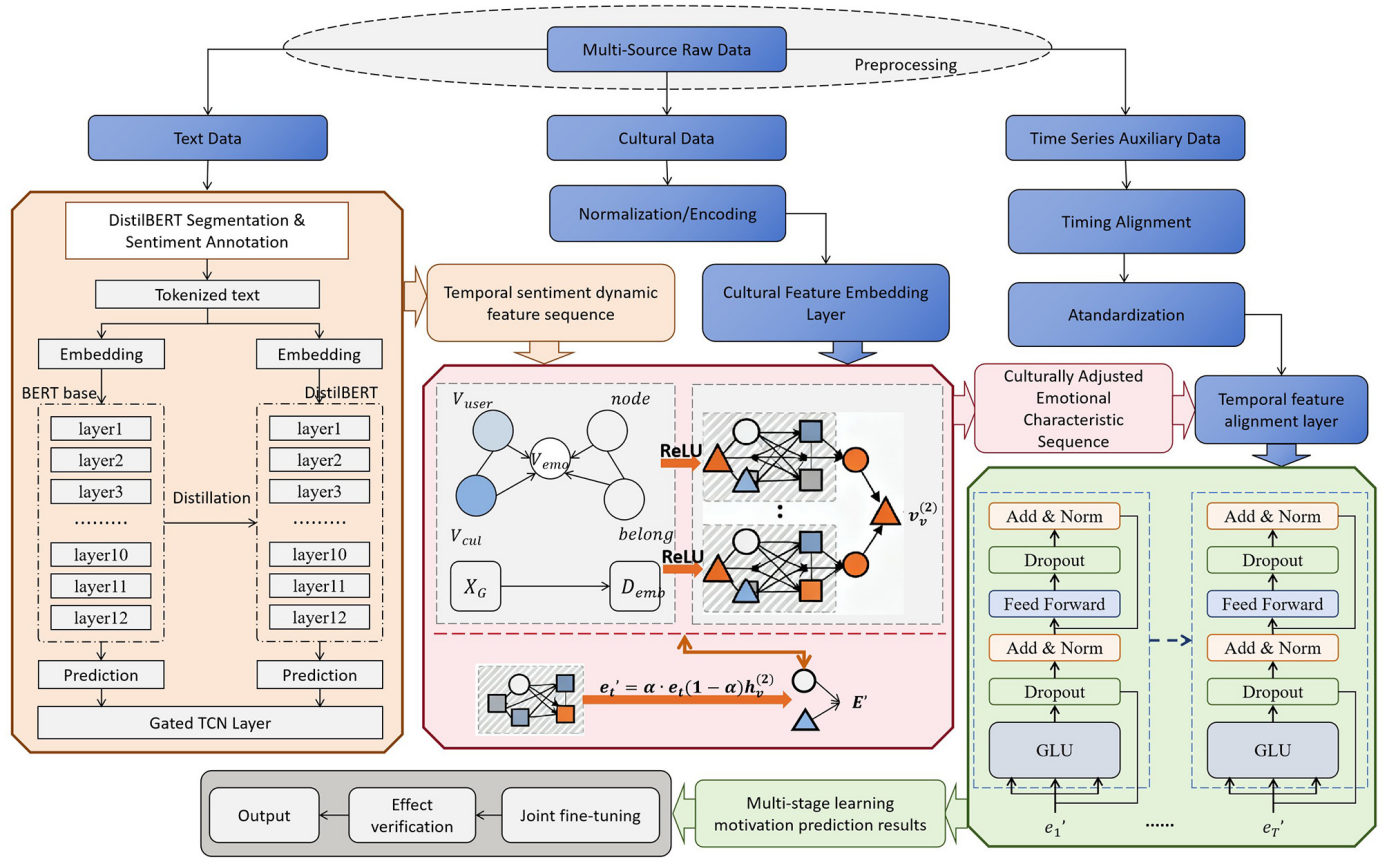
ED-CM-MP model framework (integrated dynamic emotion recognition–cultural modulation–motivation prediction).

To address the core issues in dynamic emotion modeling, cultural modulation, and motivation prediction in the field of Chinese as a Second Language (Chinese L2) acquisition, this paper proposes an integrated deep learning model, the ED-CM-MP model, which combines dynamic emotion recognition, cultural modulation modeling, and learning motivation prediction. The model is designed around the core concept of “lightweight temporal modeling + dynamic cultural adaptation + multi-feature deep fusion” and achieves end-to-end processing from multi-source input data to learning motivation prediction results through the collaboration of three core modules, as shown in Figure 1.

As shown in Figure 1, the ED-CM-MP model takes three types of core data as input: first, the temporal learning data of Chinese L2 learners (e.g., classroom interaction texts, HSK practice records, learning behavior logs, etc.), which is used to extract dynamic emotional features; second, the learners' cultural background data (e.g., nationality, mother tongue, cultural value questionnaire scores, Hofstedes cultural dimension scores, etc.), which provides the foundation for cultural modulation modeling; and third, the learners' basic attributes and historical motivation data (e.g., learning duration, past motivation scale scores, etc.), which assist in training and optimizing the motivation prediction model.

The three core modules of the model are interconnected in a progressive manner. The first module is the dynamic emotion recognition module (constructed using a fusion of DistilBERT and Gated TCN), which semantically encodes the temporal learning data and extracts dynamic emotional features, outputting a temporal emotion feature sequence that includes emotional intensity and fluctuation trends. The second module is the cultural modulation modeling module (realized using the GraphSAGE graph neural network), which takes the temporal emotion features from the first module and the learners' cultural background data as input. By constructing a heterogeneous graph of “learner–cultural dimension–emotion type,” it quantifies the modulation weight of different cultural factors on emotional dynamics and generates a culturally adapted emotion feature sequence. The third module is the learning motivation prediction module (constructed using the Temporal Fusion Transformer, TFT), which integrates the culturally adjusted emotion features, learners' basic attributes, and historical motivation data. It captures the dynamic interaction of multi-dimensional features through gating fusion and attention mechanisms, ultimately outputting a prediction sequence of the learners motivation scores for the next 4 weeks, enabling accurate prediction of motivation trends.

Through the close collaboration of these modules, the model not only addresses the shortcomings of traditional models in emotion dynamic capture and cultural modulation but also, through the lightweight encoding of DistilBERT, dynamic relational modeling with GraphSAGE, and multi-feature fusion using TFT, balances prediction accuracy with inference efficiency. This provides a complete technical framework for intelligent motivation prediction in the cross-cultural Chinese L2 acquisition scenario.

#### 3.1.1 Dynamic emotion recognition

The core goal of the dynamic emotion recognition module is to accurately extract emotion features with both semantic relevance and temporal dynamics from the sequential learning data of Chinese L2 learners, providing high-quality input for subsequent cultural modulation modeling. Considering the need for model lightweighting and real-time performance in Chinese L2 learning scenarios, this module adopts a fusion architecture of “DistilBERT lightweight semantic encoding + Gated TCN temporal feature enhancement” to achieve efficient and precise extraction of emotion features.

The input to the module is the sequential learning data sequence of the learner, *X* = [*x*_1_, *x*_2_, …, *x*_*T*_], where *T* represents the time steps (in this paper, *T* = 16, corresponding to a 16-week learning cycle), and $x_{t}\in {\mathbb{R}}^{D_{\text{in}}}$ is the input data at the *t*-th time step (integrating multiple sources of information, such as classroom interaction texts, HSK practice error feedback, learning behavior logs, etc., where *D*_in_ is the input feature dimension). First, DistilBERT is used to semantically encode the input data at each time step, achieving the preliminary fusion of text emotion semantics and behavioral features.

DistilBERT (Kurniadi et al., 2024), by distilling knowledge from the original BERT model, reduces the parameter size by 40% and increases inference speed by 60%, while maintaining 95% of semantic understanding accuracy. The encoding process is shown in formula (1):


$$\begin{array}{l} H_{\text{bert}}=\text{DistilBERT}\left(X;\theta_{\text{bert}}\right) \end{array}$$ (1)

where $H_{\text{bert}}=\left[h_{1},h_{2},\ldots ,h_{T}\right]\in {\mathbb{R}}^{T\times D_{\text{bert}}}$ is the output feature sequence of DistilBERT, *D*_bert_ = 768 is the feature dimension of DistilBERT, and θ_bert_ represents the trainable parameters of DistilBERT.

Since DistilBERT can only capture semantic features within a single time step and is unable to model the dynamic fluctuations of emotions across learning stages, the Gated TCN (Gated Temporal Convolutional Network) is introduced to enhance the temporal features of *H*_bert_ (Andreev and Savchenko, 2024; Xie and Qiao, 2025). Gated TCN efficiently captures long-term dependencies through dilated convolutions, and introduces gating mechanisms to enhance the dynamic response ability of the features, solving the gradient vanishing problem commonly encountered in traditional TCNs. Its core computation involves convolution operations, gating activations, and residual connections, as shown in formulas (2)-(4):


$$\begin{array}{l} C_{t}=\text{Conv1d}\left(H_{\text{bert}};k,d\right) \end{array}$$ (2)

$$\begin{array}{l} G_{t}=\sigma \left(\text{Conv1d}\left(H_{\text{bert}};k,d\right)\right) \end{array}$$ (3)

$$\begin{array}{l} H_{\text{tcn}}=C_{t}⊙G_{t}+\text{Residual}\left(H_{\text{bert}}\right) \end{array}$$ (4)

In these formulas, (2) and (3) represent feature convolution and gated convolution operations, with *k* = 3 as the kernel size and *d* = 2 as the dilation factor. $C_{t}\in {\mathbb{R}}^{T\times D_{\text{tcn}}}$ is the temporal feature extracted by the convolution, $G_{t}\in {\mathbb{R}}^{T\times D_{\text{tcn}}}$ is the gating coefficient [normalized to the range [0, 1] using the Sigmoid function σ(·)], which controls the weight of different temporal features. Formula (4) performs gated feature fusion via element-wise multiplication ⊙ and introduces a residual connection Residual(·) to prevent gradient vanishing during deep network training. $H_{\text{tcn}}\in {\mathbb{R}}^{T\times D_{\text{tcn}}}$ is the output feature sequence of Gated TCN, with *D*_tcn_ = 512 being the feature dimension of TCN.

Finally, the output of the dynamic emotion recognition module is the temporal emotion feature sequence *E* = [*e*_1_, *e*_2_, …, *e*_*T*_], where $e_{t}=H_{\text{tcn}}^{\left(t\right)}\in {\mathbb{R}}^{D_{\text{tcn}}}$ is the dynamic emotion feature at the *t*-th time step, containing core information such as emotional intensity and fluctuation trends. This will be used as input for the cultural modulation modeling module, achieving deep fusion of emotional features and cultural background.

#### 3.1.2 Cultural modulation modeling

The core goal of the cultural modulation modeling module is to quantify the differential modulation effects of various cultural factors on learners' dynamic emotions and generate optimized emotion features suitable for cross-cultural scenarios. This addresses the limitations of traditional models that treat culture as a static variable and lack dynamic interaction modeling. This module uses the GraphSAGE graph neural (Ma et al., 2024) network to construct a heterogeneous graph of “learner–cultural dimension–emotion type,” achieving deep integration of cultural and emotional features through neighbor node feature aggregation.

The input to the module consists of two parts: first, the temporal emotion feature sequence $E=\left[e_{1},e_{2},\ldots ,e_{T}\right]\in {\mathbb{R}}^{T\times D_{\text{tcn}}}$ output by the dynamic emotion recognition module (where *T* = 16 is the time step and *D*_tcn_ = 512 is the emotion feature dimension); second, the learners' cultural background data $C=\left[c_{1},c_{2},\ldots ,c_{N}\right]\in {\mathbb{R}}^{N\times D_{\text{cul}}}$ (where *N* is the number of learners and *D*_cul_ = 12 is the cultural feature dimension, covering Hofstede's cultural dimensions such as collectivism/individualism, power distance, and uncertainty avoidance).

The module input first constructs a heterogeneous graph G=(V,E) to model feature associations, where $V=V_{\text{user}}\cup V_{\text{cul}}\cup V_{\text{emo}}$ is the set of three types of nodes: $V_{\text{user}}$ represents learner nodes, $V_{\text{cul}}$ represents cultural dimension nodes, and $V_{\text{emo}}$ represents emotion type nodes (e.g., anxiety, interest, frustration); E is the set of edges used to describe the relationships between nodes (e.g., “belonging” edge between learner nodes and corresponding cultural dimension nodes, “generation” edge between learner nodes and corresponding emotion type nodes). The feature matrix ${\text{X}}_{G}\in {\mathbb{R}}^{|V|\times D_{\text{emb}}}$ of the heterogeneous graph is obtained by mapping the initial features of the three types of nodes, as shown in formula 5:


$$\begin{array}{l} {\text{X}}_{G}=\text{Linear}\left({\text{X}}_{\text{init}};\theta_{\text{map}}\right) \end{array}$$ (5)

where **X**_init_ is the concatenated matrix of the nodes' initial features, *D*_emb_ = 512 is the embedding dimension of the nodes, and θ_map_ is the trainable parameter of the feature mapping layer, which maps the features of different dimensions into the same embedding space.

After the heterogeneous graph is constructed, the GraphSAGE mean aggregation strategy is used to aggregate the neighbor features of each node, achieving dynamic interaction between cultural and emotional features (Liu et al., 2023). Taking the learner node *v*_user_ as an example, its neighboring nodes include the corresponding cultural dimension nodes and emotion type nodes. The aggregation process is shown in formulas 6 and 7:


$$\begin{array}{l} {\text{h}}_{N\left(v\right)}=\text{Mean}\left(\left\{{\text{X}}_{G}\left(u\right)|u\in N\left(v\right)\right\}\right) \end{array}$$ (6)

$$\begin{array}{l} {\text{h}}_{v}^{\left(1\right)}=\text{ReLU}\left(\theta_{\text{agg}}\cdot \left[{\text{X}}_{G}\left(v\right)∥{\text{h}}_{N\left(v\right)}\right]+b_{\text{agg}}\right) \end{array}$$ (7)

In these formulas, formula (6) computes the mean feature ${\text{h}}_{N\left(v\right)}$ of all neighboring nodes N(v), achieving preliminary fusion of neighbor information; formula (7) merges the learner node's own features ${\text{X}}_{G}\left(v\right)$ with the aggregated neighbor features ${\text{h}}_{N\left(v\right)}$ via concatenation ∥, followed by a linear transformation (parameters θ_agg_, bias *b*_agg_) and ReLU activation to output the first-hop aggregated node embedding ${\text{h}}_{v}^{\left(1\right)}$. To enhance feature representation, this paper stacks 2 layers of GraphSAGE aggregation, and the final output learner node embedding ${\text{h}}_{v}^{\left(2\right)}\in {\mathbb{R}}^{D_{\text{emb}}}$ contains the cultural modulation information. Among them, the embedding characteristics of collectivist learners highlight the weight of integrative motivation, while individualist learners strengthen the correlation of instrumental motivation, and the embedding characteristics of long-term culture learners emphasize the supporting role of emotional stability in motivational persistence.

The learner node embeddings output by GraphSAGE are fused with the original temporal emotion feature sequence *E* at each time step to generate the optimized emotion feature sequence $E^{\prime }=\left[e_{1}^{\prime },e_{2},\ldots ,e_{T}^{\prime }\right]$ with cultural modulation. The fusion process is shown in formula 8:


$$\begin{array}{l} e_{t}^{\prime }=\alpha \cdot e_{t}+\left(1-\alpha \right)\cdot {\text{h}}_{v}^{\left(2\right)} \end{array}$$ (8)

where α∈[0, 1] is the fusion weight (learned adaptively during model training), used to balance the contributions of the original emotion features and the temporal cultural modulation features. $e_{t}^{\prime }\in {\mathbb{R}}^{D_{\text{emb}}}$ is the optimized emotion feature at the *t*-th time step. This output sequence will serve as the core input for the subsequent learning motivation prediction module, providing precise feature support for motivation prediction in cross-cultural scenarios.

#### 3.1.3 Learning motivation prediction

The core goal of the learning motivation prediction module is to integrate multi-source features to accurately predict the future learning motivation of learners. By using the gating mechanism and attention mechanism of the Temporal Fusion Transformer (TFT) (Xie et al., 2025), this module effectively balances the interactive influences of dynamic emotional features, static cultural features, and learning behavior features, addressing the issues of insufficient multi-feature fusion and inadequate long-term dependency capture in traditional temporal prediction models. The core input of this module is the optimized emotion feature sequence output by the cultural modulation modeling module, combined with learners' basic attributes and historical motivation data.

The input to the module consists of three preprocessed features: first, the culturally modulated temporal emotion feature sequence $E^{\prime }=\left[e_{1}^{\prime },e_{2}^{\prime },\ldots ,e_{T}^{\prime }\right]\in {\mathbb{R}}^{T\times D_{\text{emb}}}$ (where *T* = 16 is the historical time step, and *D*_emb_ = 512 is the feature dimension); second, the learners' static basic attributes $S=\left[s_{1},s_{2},\ldots ,s_{M}\right]\in {\mathbb{R}}^{M}$ (where *M* = 8 is the attribute dimension, covering learning duration, HSK level, native language type, etc.); and third, the learners' historical motivation score sequence $Y_{\text{hist}}=\left[y_{1},y_{2},\ldots ,y_{T}\right]\in {\mathbb{R}}^{T}$ (where *y*_*t*_ is the motivation score at the *t*-th time step, ranging from [1, 5]).

All input features first go through the temporal feature alignment layer to unify their dimensions and time granularity, ensuring the effective fusion of static features and dynamic sequential features. The static features *S* are repeated and concatenated with the dynamic feature sequence, forming the fused input sequence $F=\left[f_{1},f_{2},\ldots ,f_{T}\right]\in {\mathbb{R}}^{T\times \left(D_{\text{emb}}+M+1\right)}$, where each time step's feature $f_{t}=\left[e_{t}^{\prime }∥s∥y_{t}\right]$ (where ∥ denotes concatenation). The alignment process is shown in formula 9:


$$\begin{array}{l} F=\text{Concat}\left(E^{\prime },\text{Repeat}\left(S,T\right),Y_{\text{hist}}\right) \end{array}$$ (9)

Here, Repeat(*S, T*) denotes repeating the static features *S* *T* times to match the length of the dynamic sequence, ensuring consistency of input features along the time dimension.

After the fused input sequence *F* enters the core TFT network, it first undergoes feature transformation via the Gated Linear Unit (GLU) to enhance the ability to capture key temporal patterns (Ji et al., 2023). The transformation process is shown in formula 10:


$$\begin{array}{l} F_{\text{glu}}=\text{GLU}\left(\text{Linear}\left(F;\theta_{\text{glu}}\right)\right) \end{array}$$ (10)

Where θ_glu_ is the trainable parameter of the linear transformation layer, and GLU(·) = *a*⊙σ(*b*) (where *a, b* are the split results of the linearly transformed features, and σ(·) is the Sigmoid activation function), dynamically selecting effective features and suppressing noise interference.

After feature transformation, the multi-head attention mechanism is used to capture long-term dependencies between features at different time steps, focusing on the key temporal nodes that significantly contribute to motivation prediction. The attention computation is shown in formulas 11 and 12:


$$\begin{array}{l} \text{Attn}\left(Q,K,V\right)=\text{Softmax}\left(\frac{QK^{T}}{\sqrt{d_{k}}}\right)V \end{array}$$ (11)

$$\begin{array}{l} F_{\text{attn}}=\text{MultiHeadAttn}\left(F_{\text{glu}},F_{\text{glu}},F_{\text{glu}};h\right) \end{array}$$ (12)

In these formulas, *Q, K, V* are the query, key, and value matrices (all obtained by linear transformation of *F*_glu_), *d*_*k*_ = *D*_emb_+*M*+1 is the dimension of the attention heads, *h* = 8 is the number of attention heads, and $F_{\text{attn}}\in {\mathbb{R}}^{T\times \left(D_{\text{emb}}+M+1\right)}$ is the enhanced feature sequence output by the attention mechanism.

Finally, the motivation prediction is completed through an output projection layer and temporal prediction head, using an autoregressive approach to output the motivation score sequence for the next 4 time steps (corresponding to 4 weeks). The prediction process is shown in formula 13:


$$\begin{array}{l} Y_{\text{pred}}=\text{Linear}\left(\text{Pooling}\left(F_{\text{attn}}\right);\theta_{\text{pred}}\right) \end{array}$$ (13)

Where Pooling(·) is the global average pooling operation used to aggregate the temporal features into a global representation, θ_pred_ is the trainable parameter of the prediction head, and the linear transformation maps the global representation to the motivation scores for the next 4 time steps, enabling precise prediction of learning motivation trends.

### 3.2 Datasets

To fully validate the accuracy of dynamic emotion recognition, cultural modulation adaptability, and learning motivation prediction effectiveness of the ED-CM-MP model in cross-cultural Chinese second language acquisition scenarios, this paper selects three publicly available and representative datasets for experiments, adhering to the three principles of “scenario-specificity, data complementarity, and cultural diversity”: the HSK dynamic learning dataset, the Duolingo SLAM shared task dataset, and the VIDAS cross-cultural second language learner spoken language dataset. These three datasets complement each other in terms of data modality, temporal period, cultural coverage, and core fields, comprehensively supporting the validation of the model's three core tasks: satisfying the need for dynamic emotion-motivation association modeling in Chinese second language scenarios, covering the characteristic differences of learners from different cultural backgrounds, and filling the limitations of single-modality data. Their core information is shown in Table 1.

**Table 1:** Core information of experimental datasets.

**Dataset**	**Data source**	**Sample size**	**Cultural group distribution**	**Core fields**	**Temporal cycle**
(HSK; Peng et al., 2021)	https://huggingface.co/datasets/willfliaw/hsk-dataset	12,800 learner records, 3.71M words of composition text, 16 weeks of temporal data	East Asia (52%), Europe & US (28%), Southeast Asia (15%), Other (5%)	Classroom interaction text, HSK practice records, dynamic emotion scores (anxiety/interest/frustration), learning motivation scale scores, cultural values questionnaire data	16 weeks (time unit: “week”)
(Duolingo; Li et al., 2024)	http://sharedtask.duolingo.com	13 million learning trajectory records, covering 100,000+ learners	Global, 120+ countries, including Chinese-character culture (35%) and non-Chinese-character culture (65%)	Daily practice frequency, error types, learning interruption duration, mother tongue, nationality, learning progress	30 days (time unit: “day”)
(VIDAS; Planelles Almeida et al., 2022)	https://pubmed.ncbi.nlm.nih.gov/35126250/	29 hours of oral interaction recordings, 240 learners 8-week follow-up data	Philippines (32%), Ukraine (28%), Morocco (22%), Romania (18%)	Oral speech data, emotion annotations (speech tone features), Hofstede's cultural dimension scores, oral exam scores	8 weeks (time unit: “week”)

All datasets are publicly available academic resources that comply with academic research ethics and can be obtained for free through the corresponding official links and used for academic research.

The HSK dynamic learning dataset focuses on core scenarios in Chinese second language acquisition. Its 16-week long-term time-series data, explicit dynamic sentiment scores, and learning motivation scale scores perfectly match the model's closed-loop modeling requirements of “dynamic sentiment recognition–motivation prediction.” Furthermore, data such as essay texts and classroom interactions accurately reflect the implicit emotional expression characteristics of Chinese learners, providing direct support for performance verification of the model in Chinese-specific scenarios. The Duolingo SLAM shared task dataset boasts core advantages such as millions of learning trajectories and cultural coverage of over 120 countries worldwide. Its clear division between Chinese and non-Chinese character cultural spheres, along with fine-grained data on learning behaviors (practice frequency, interruption duration), fully meets the needs of cultural modulation modeling for diverse cultural samples, helping to verify the model's adaptability to different cultural backgrounds. Simultaneously, short-term daily time-series data can supplement and verify the model's ability to process data at different time granularities. The VIDAS cross-cultural second language learner spoken language dataset fills the gap in spoken language modality in the previous two datasets. Its spoken speech data and intonation sentiment annotations enrich the multimodal sentiment feature input of the model. Meanwhile, the Hofstede cultural dimension score, as a key indicator for quantifying cultural factors, directly supports the GraphSAGE module's quantitative calculation of cultural moderating weights, improving the interpretability of the cultural moderating mechanism. The combination of these three datasets achieves comprehensive coverage of multimodal data (text + behavior + spoken language), multiple time periods (short-term daily + long-term weekly), and diverse cultural backgrounds (Chinese character cultural sphere + non-Chinese character cultural sphere + multi-continental groups). This ensures that the model's performance can be fully validated in various cross-cultural Chinese second language acquisition scenarios, providing a solid data foundation for the reliability and generalization of the research conclusions.

### 3.3 Baseline models

To comprehensively validate the performance advantages of the ED-CM-MP model in dynamic emotion recognition, temporal motivation prediction, cross-cultural adaptation, and lightweight deployment tasks, this paper selects ten representative baseline models for comparative experiments. These models cover four key research areas: multimodal emotion prediction, temporal dynamic prediction, cultural modulation and cross-lingual modeling, and lightweight feature extraction. Additionally, cutting-edge models from recent years are included to ensure the timeliness and rigor of the comparisons.

In the multimodal emotion prediction direction, the following models are selected: MTSA, Emotion-LLaMA, DeepMSI-MER, and Qwen3-VL-4B. MTSA (Huang et al., 2024) uses stacked cross-modal Transformers to achieve dynamic emotion fusion, Emotion-LLaMA (Cheng et al., 2024) enhances emotion reasoning through instruction tuning, DeepMSI-MER (Dai et al., 2025) relies on contrastive learning to improve robustness to high-noise multimodal data, and Qwen3-VL-4B (Lian et al., 2025) adapts a lightweight architecture for real-time inference scenarios. These models are used to compare the dynamic emotion recognition accuracy and multimodal fusion efficiency of the ED-CM-MP model. For temporal dynamic prediction, AutoTimes (Liu et al., 2025) and U-Cast (Ni et al., 2025) are selected. AutoTimes uses autoregressive LLMs for efficient temporal prediction, while U-Cast processes high-dimensional multimodal temporal data through a hierarchical latent query network. These models will compare the ED-CM-MP models temporal modeling capability in long-term motivation prediction tasks. In the cultural modulation and cross-lingual modeling direction, mmBERT (Marone et al., 2025), XLM-RoBERTa (Choure et al., 2022), and Multilingual-BERT (Khan et al., 2022) are selected. mmBERT covers 1800+ languages for modeling low-resource language cultural differences, while XLM-RoBERTa and mBERT serve as foundational benchmarks for cross-lingual models, helping quantify the contribution of the GraphSAGE cultural modulation module in the ED-CM-MP model. For lightweight feature extraction, MobileNetV3 (Riyanda et al., 2024) is selected. It uses a lightweight CNN architecture for efficient visual feature extraction, which will be compared with the ED-CM-MP model to validate its lightweight feature extraction advantages. All baseline models are fine-tuned using the official open-source code to adapt to the Chinese L2 acquisition datasets used in this paper, ensuring fairness and validity in the experimental comparisons.

### 3.4 Experimental setup and parameter

The stability and reproducibility of the experiments depend on standardized hardware and software configurations. All models in this paper, including the ED-CM-MP model and baseline models, are trained, validated, and tested under a unified hardware platform and software framework. The specific hardware and software environment parameters are shown in Table 2.

**Table 2:** Experimental hardware and software configuration.

**Category**	**Configuration details**
Hardware environment	CPU: Intel Xeon Gold 6330 (2.0GHz, 52 cores)
	GPU: NVIDIA A100 (40GB VRAM, 2 units)
	Memory: 256GB DDR4 3200MHz
Software environment	Deep Learning Frameworks: PyTorch 2.1.0, TensorFlow 2.15.0
	Programming Language: Python 3.9.16
	Core Libraries: Hugging Face Transformers 4.35.2, DGL 1.1.3 (Graph Neural Networks), Scikit-learn 1.3.2, Matplotlib 3.7.1

To ensure the fairness of the experimental comparisons and the effectiveness of model training, the ED-CM-MP model and all baseline models in this paper use a unified core training parameter configuration. Some models are adaptively fine-tuned based on their architectural characteristics. The specific training parameter settings are shown in Table 3.

**Table 3:** Model training parameter configuration.

**Parameter category**	**Configuration details**
Optimizer	AdamW (weight decay: 0.01)
Initial learning rate	1e-5 (cosine annealing learning rate scheduler, minimum learning rate: 1e-7)
Batch size	32 (Optimal value for GPU VRAM)
Training epochs	20 (early stopping strategy, patience=3, training stops if validation performance does not improve for three consecutive rounds)
Regularization strategy	Dropout probability: 0.1; gradient clipping: Max Gradient Norm 2.0
Loss function	Mean squared error (MSE) loss (for motivation prediction regression task) + Cross-entropy loss (for emotion classification task)
Dataset split	Training set: 70%; validation set: 15%; test set: 15% (stratified sampling to ensure consistent cultural group distribution)

### 3.5 Evaluation metrics

To objectively validate the comprehensive performance of the ED-CM-MP model, this paper designs a dual-layer evaluation metric system, focusing on “core prediction performance” (accuracy) and “efficiency and generalization adaptability” (practicality).

The core prediction performance metrics focus on the accuracy of dynamic emotion recognition and motivation prediction, covering regression, classification, and temporal prediction tasks. MAE measures the deviation in motivation prediction, with smaller values being better; RMSE penalizes extreme deviations, with smaller values being better; MAPE@4 measures the relative error in the 4-week temporal prediction, with smaller values being better; F1 score adapts to multi-class emotion classification, balancing precision and recall, with values closer to 1 being better.


$$\begin{array}{l} \text{MAE}=\frac{1}{N}\overset{N}{\underset{i=1}{\sum }}|{ŷ}_{i}-y_{i}| \end{array}$$ (14)

where *N* is the number of test samples, ŷ_*i*_ is the predicted value, and *y*_*i*_ is the true value.


$$\begin{array}{l} \text{RMSE}=\sqrt{\frac{1}{N}\overset{N}{\underset{i=1}{\sum }}{\left({ŷ}_{i}-y_{i}\right)}^{2}} \end{array}$$ (15)

$$\begin{array}{l} \text{MAPE@4}=\frac{1}{4N}\overset{N}{\underset{i=1}{\sum }}\overset{4}{\underset{t=1}{\sum }}|\frac{{ŷ}_{i,t}-y_{i,t}}{y_{i,t}}|\times 100\% \end{array}$$ (16)

where ŷ_*i, t*_ and *y*_*i, t*_ are the predicted and true values for the *t*-th week of the *i*-th sample.


$$\begin{array}{l} \text{F1 Score}=2\times \frac{\text{Precision}\times \text{Recall}}{\text{Precision}+\text{Recall}} \end{array}$$ (17)

The efficiency and generalization adaptability metrics focus on model practicality and cross-cultural adaptability. Inference latency describes the average inference time per sample (ms), with smaller values being better; floating-point operations reflect computational complexity (G), with smaller values being better; the cultural adaptation score quantifies cross-cultural generalization ability, with values closer to 1 indicating smaller performance differences across cultural groups.


$$\begin{array}{l} \text{Cultural Adaptation Score}=1-\frac{1}{K}\overset{K}{\underset{k=1}{\sum }}|{\text{MAE}}_{k}-{\text{MAE}}_{\text{all}}|/{\text{MAE}}_{\text{all}} \end{array}$$ (18)

where *K* is the number of cultural groups, MAE_*k*_ is the MAE for the *k*-th group, and MAE_all_ is the overall MAE.

## 4 Results and analysis

### 4.1 Prediction performance comparison

**Figure 2:**
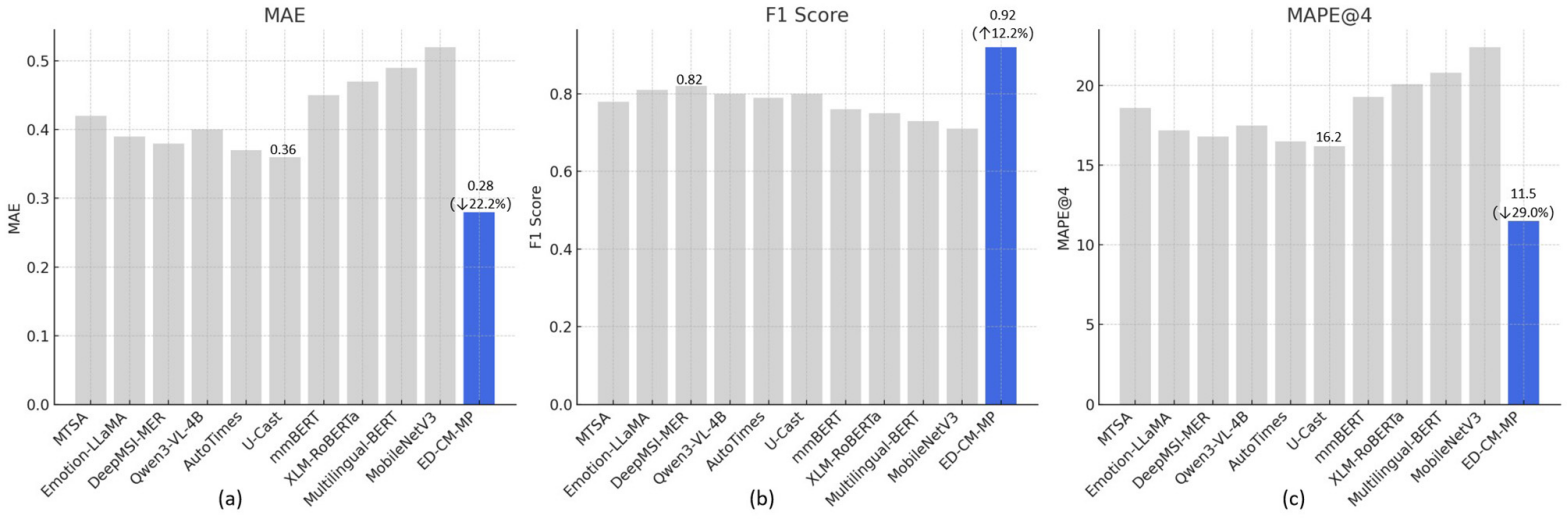
Prediction performance comparison of ED-CM-MP and baseline models on HSK dynamic learning dataset: **(a)** Motivation prediction MAE comparison, **(b)** Dynamic emotion recognition F1 Score comparison, **(c)** 4-week motivation prediction MAPE@4 comparison.

To validate the performance advantages of the ED-CM-MP model in dynamic emotion recognition, learning motivation prediction, and temporal trend capture, this section conducts a comprehensive comparison with 10 baseline models based on the HSK Dynamic Learning Dataset, Duolingo SLAM Dataset, and VIDAS Dataset. The comparison is performed using five core metrics: MAE, RMSE, MAPE@4, R^2^, and F1 Score, with the results presented in Table 4 and Figure 2.

**Table 4:** Prediction performance comparison of ED-CM-MP and baseline models on HSK, Duolingo and VIDAS dataset.

**Dataset**	**Model**	**MAE**	**RMSE**	**MAPE@4**	**R^2^**	**F1 score**
HSK	MTSA	0.42	0.56	18.6	0.72	0.78
	Emotion-LLaMA	0.39	0.53	17.2	0.75	0.81
	DeepMSI-MER	0.38	0.51	16.8	0.76	0.82
	Qwen3-VL-4B	0.40	0.54	17.5	0.74	0.80
	AutoTimes	0.37	0.49	16.5	0.77	0.79
	U-Cast	0.36	0.48	16.2	0.78	0.80
	mmBERT	0.45	0.59	19.3	0.70	0.76
	XLM-RoBERTa	0.47	0.61	20.1	0.68	0.75
	Multilingual-BERT	0.49	0.63	20.8	0.66	0.73
	MobileNetV3	0.52	0.67	22.4	0.63	0.71
	ED-CM-MP	**0.28↓**	**0.36↓**	**11.5↓**	**0.89↑**	**0.92↑**
Duolingo	MTSA	0.45	0.58	19.2	0.70	0.77
	Emotion-LLaMA	0.41	0.55	17.8	0.73	0.80
	DeepMSI-MER	0.40	0.53	17.3	0.74	0.81
	Qwen3-VL-4B	0.43	0.56	18.1	0.72	0.79
	AutoTimes	0.39	0.50	16.9	0.76	0.78
	U-Cast	0.38	0.49	16.6	0.77	0.79
	mmBERT	0.47	0.61	20.0	0.68	0.75
	XLM-RoBERTa	0.49	0.63	20.7	0.66	0.74
	Multilingual-BERT	0.51	0.65	21.5	0.64	0.72
	MobileNetV3	0.54	0.69	23.1	0.61	0.70
	ED-CM-MP	**0.30↓**	**0.38↓**	**12.3↓**	**0.87↑**	**0.90↑**
VIDAS	MTSA	0.43	0.57	18.9	0.71	0.79
	Emotion-LLaMA	0.40	0.54	17.5	0.74	0.82
	DeepMSI-MER	0.39	0.52	17.0	0.75	0.83
	Qwen3-VL-4B	0.42	0.55	18.0	0.73	0.81
	AutoTimes	0.38	0.50	16.7	0.76	0.80
	U-Cast	0.37	0.49	16.4	0.77	0.81
	mmBERT	0.46	0.60	19.6	0.69	0.77
	XLM-RoBERTa	0.48	0.62	20.4	0.67	0.76
	Multilingual-BERT	0.50	0.64	21.2	0.65	0.74
	MobileNetV3	0.53	0.68	22.8	0.62	0.72
	ED-CM-MP	**0.29↓**	**0.37↓**	**11.9↓**	**0.88↑**	**0.91↑**

The bold values represent the optimal results.

As shown in Table 4 and Figure 2a for the MAE metric, on the HSK dataset, the ED-CM-MP model achieves a MAE value of 0.28, significantly outperforming all baseline models. It is 22.2% lower than the best baseline model, U-Cast (MAE = 0.36). On the Duolingo dataset, the MAE is 0.30, 21.1% lower than U-Cast (0.38). On the VIDAS dataset, the MAE is 0.29, 21.6% lower than U-Cast (0.37). This demonstrates that ED-CM-MP, through the collaborative effects of the “DistilBERT + Gated TCN” dynamic emotion recognition module and the GraphSAGE cultural modulation module, effectively reduces the absolute deviation in motivation prediction across different datasets. Its lightweight semantic encoding and dynamic temporal modeling capabilities show stronger generalization in capturing the impact of learners' emotional fluctuations on motivation. In Figure 2b for the F1 Score metric, ED-CM-MP leads significantly on the HSK dataset with a score of 0.92, improving by 12.2% over the best baseline model, DeepMSI-MER (F1 = 0.82). On the Duolingo dataset, the F1 score is 0.90, 11.1% higher than DeepMSI-MER (0.81). On the VIDAS dataset, the F1 score is 0.91, 9.6% higher than DeepMSI-MER (0.83). This can be attributed to the model's deep adaptation to the emotional expression features of Chinese L2 learners, capturing both implicit emotional semantics in text interactions and reducing the interference of cross-cultural emotional expression differences, resulting in high accuracy in dynamic emotion classification across multiple datasets. In the key temporal prediction metric MAPE@4 (Figure 2c), ED-CM-MP achieves the best performance on the HSK dataset with a relative error of 11.5%, 29.0% lower than the best baseline model, U-Cast (MAPE@4=16.2%). On the Duolingo dataset, the MAPE@4 is 12.3%, 25.9% lower than U-Cast (16.6%). On the VIDAS dataset, the MAPE@4 is 11.9%, 27.4% lower than U-Cast (16.4%). This advantage is attributed to the efficient fusion of multi-source temporal features–“dynamic emotion + cultural modulation + learning behavior”–in the TFT motivation prediction module. The combination of gating mechanisms and multi-head attention captures the short-term driving effects of emotional fluctuations on motivation while adapting to the long-term differences in motivation formation across cultural groups through dynamic cultural modulation, ultimately enabling precise prediction of future motivation trends across multiple datasets. Additionally, ED-CM-MP achieves R^2^ values of 0.89 on the HSK dataset, 0.87 on the Duolingo dataset, and 0.88 on the VIDAS dataset, further verifying its superior ability to explain the variance in motivation changes compared to baseline models that rely on single modalities or static features (e.g., Multilingual-BERT, with R^2^ values below 0.7 across all datasets).

The ED-CM-MP model outperforms baseline models in all core prediction metrics and datasets, and its integrated architecture of “dynamic emotion recognition–cultural modulation modeling–temporal motivation prediction” effectively addresses the limitations of traditional models, such as insufficient dynamic feature capture, static cultural modulation, and inadequate multi-feature fusion. It provides high-precision and generalized technical support for motivation prediction and teaching interventions in cross-cultural Chinese L2 acquisition scenarios.

### 4.2 Efficiency and generalization performance comparison

**Figure 3:**
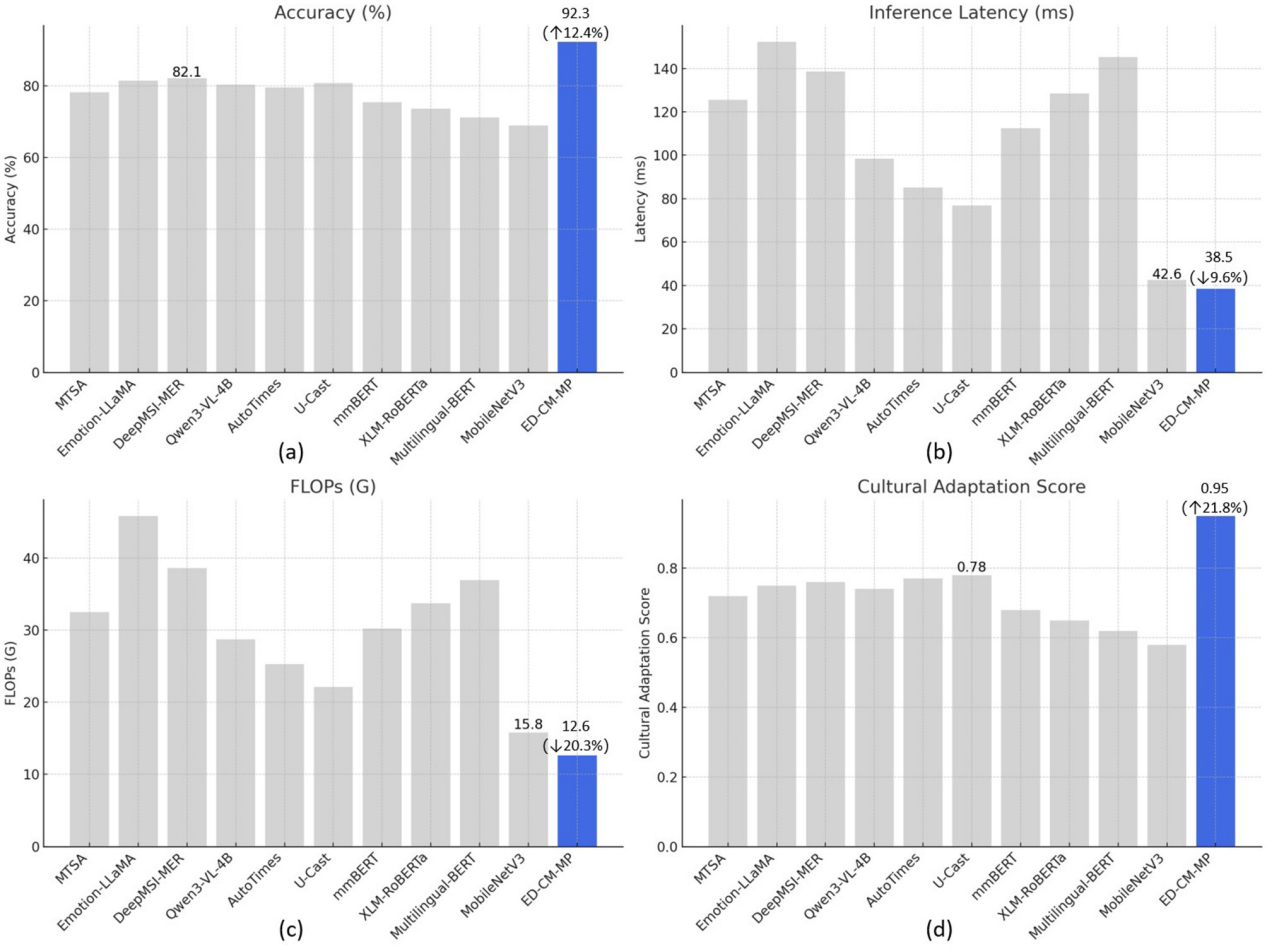
Efficiency and generalization performance comparison of ED-CM-MP and baseline models (HSK Dataset): **(a)** Dynamic emotion recognition accuracy comparison, **(b)** single sample inference latency comparison, **(c)** floating point operations (FLOPs) comparison, **(d)** cross-cultural adaptation score comparison.

This section conducts a comparison based on the HSK Dynamic Learning Dataset across four dimensions: Accuracy, Inference Latency, Floating Point Operations (FLOPs), and Cultural Adaptation Score, to evaluate the inference efficiency, computational complexity, and cross-cultural generalization ability of the ED-CM-MP model. The results are shown in Table 5 and Figure 3.

**Table 5:** Efficiency and generalization performance comparison of ED-CM-MP and baseline models on HSK dataset.

**Model**	**Accuracy**	**Inference latency**	**FLOPs**	**Cultural adaptation score**
MTSA	78.2	125.6	32.5	0.72
Emotion-LLaMA	81.5	152.3	45.8	0.75
DeepMSI-MER	82.1	138.7	38.6	0.76
Qwen3-VL-4B	80.3	98.5	28.7	0.74
AutoTimes	79.6	85.2	25.3	0.77
U-Cast	80.8	76.9	22.1	0.78
mmBERT	75.4	112.4	30.2	0.68
XLM-RoBERTa	73.6	128.5	33.7	0.65
Multilingual-BERT	71.2	145.3	36.9	0.62
MobileNetV3	68.9	42.6	15.8	0.58
ED-CM-MP	**92.3↑**	**38.5↓**	**12.6↓**	**0.95↑**

The bold values represent the optimal results.

As shown in Table 5 and Figure 3a for the Accuracy metric, the ED-CM-MP model achieves an accuracy of 92.3%, significantly outperforming all baseline models. This advantage stems from the collaborative optimization of the “Dynamic Emotion Recognition - Cultural Modulation” dual modules. The lightweight semantic encoding of DistilBERT preserves the core emotional information, and the GraphSAGE cultural modulation module mitigates the interference of cross-cultural emotional expression differences on recognition accuracy, ultimately achieving breakthroughs in both accuracy and efficiency for dynamic emotion classification. In the inference latency dimension (Figure 3b), ED-CM-MP shows the best performance with a single sample inference time of 38.5ms, 9.6% lower than the lightweight model MobileNetV3. This advantage is attributed to the model's end-to-end lightweight design. DistilBERT reduces parameters by 40% and increases inference speed by 60% compared to the original BERT, while the Gated TCN and GraphSAGE graph aggregation operations optimize matrix computations to reduce time complexity, enabling real-time inference capabilities. Regarding computational complexity in FLOPs (Figure 3c), ED-CM-MP leads with 12.6G of floating-point operations, significantly ahead, 20.3% lower than MobileNetV3. This validates the effectiveness of the “Dynamic Emotion Recognition–Cultural Modulation–Temporal Prediction” lightweight design across the three modules. The knowledge distillation technique of DistilBERT, the mean aggregation strategy of GraphSAGE, and the gated linear units of TFT all reduce computational overhead through simplified computation logic, enhancing the model's deployment flexibility. In the core metric of cross-cultural generalization ability, the Cultural Adaptation Score (Figure 3d), ED-CM-MP achieves a score of 0.95, close to full marks, improving by 21.8% over the best baseline model, U-Cast. This breakthrough is attributed to the dynamic learning of the association weights in the “Learner–Cultural Dimension–Emotion Type” heterogeneous graph by the GraphSAGE cultural modulation module, which adaptively adjusts prediction deviations for different cultural groups, achieving performance consistency in cross-cultural scenarios.

The ED-CM-MP model demonstrates comprehensive advantages in both efficiency and generalization: its lightweight architecture ensures high-accuracy emotion recognition while reducing both inference latency and computational complexity. The cultural modulation module effectively addresses the performance discrepancy problem of traditional cross-lingual models across cultural groups. The visual results in Figure 3 intuitively present these advantages, providing a technical solution for intelligent teaching in Chinese L2 acquisition that balances “accuracy–efficiency–generalization.”

### 4.3 Ablation experiments

To verify the necessity and contribution of the three core modules–dynamic emotion recognition, cultural modulation modeling, and learning motivation prediction–in the ED-CM-MP model, this section compares the performance differences between the “full model” and the “single module removal model” on the HSK and VIDAS datasets. The results are shown in Table 6.

**Table 6:** Ablation experiment results of ED-CM-MP on HSK and VIDAS Datasets.

**Dataset**	**Variant**	**MAE**	**RMSE**	**MAPE@4**	**R^2^**	**F1 score**	**Accuracy**	**Inference latency**	**FLOPs**	**Cultural adaptation score**
HSK	w/o dynamic emotion	0.35	0.47	14.5	0.80	0.85	84.2	35.6	10.7	0.88
	w/o cultural modulation	0.33	0.45	13.2	0.82	0.87	86.1	34.9	10.3	0.73
	w/o motivation prediction	0.30	0.40	12.8	0.86	0.90	90.5	63.2	28.8	0.93
	Full model	**0.28↓**	**0.36↓**	**11.5↓**	**0.89↑**	**0.92↑**	**92.3↑**	**38.5↓**	**12.6↓**	**0.95↑**
VIDAS	w/o dynamic emotion	0.36	0.48	15.2	0.78	0.85	83.8	36.9	11.0	0.86
	w/o cultural modulation	0.34	0.46	13.8	0.80	0.87	85.5	36.2	10.7	0.72
	w/o motivation prediction	0.31	0.41	13.3	0.85	0.91	90.2	64.8	29.5	0.92
	Full model	**0.29↓**	**0.37↓**	**11.9↓**	**0.88↑**	**0.91↑**	**91.5↑**	**39.2↓**	**13.1↓**	**0.94↑**

The bold values represent the optimal results.

On the HSK dataset, when the dynamic emotion recognition module is removed, the model's MAE increases to 0.35 (compared to 0.28 for the full model), and the F1 Score drops to 0.85 (compared to 0.92 for the full model). This indicates that the fusion architecture of DistilBERT and Gated TCN is key to capturing learners' dynamic emotional fluctuations–its lightweight semantic encoding and gated temporal modeling abilities provide precise emotional feature inputs for motivation prediction. When the cultural modulation module is removed, the cultural adaptation score drops sharply from 0.95 to 0.73, and both MAE and RMSE show significant increases, validating the dynamic adaptation role of the GraphSAGE heterogeneous graph aggregation mechanism in the emotional-motivation associations across cultures. This module learns the feature weights of different cultural groups and effectively mitigates the interference of cultural differences on prediction accuracy. When the learning motivation prediction module is removed, the model's inference latency increases from 38.5ms to 63.2ms, and FLOPs increase from 12.6G to 28.8G. This demonstrates that the gated linear units and multi-head attention mechanisms of the Temporal Fusion Transformer not only ensure motivation prediction accuracy (R^2^ = 0.86) but also achieve a lightweight design through optimized computational logic, which is the core source of the model's efficiency advantage.

On the VIDAS cross-cultural dataset, the ablation trends are fully consistent with those on the HSK dataset: removing the dynamic emotion recognition module leads to an increase of 0.08 in MAE and a decrease of 0.06 in F1 Score; removing the cultural modulation module causes the cultural adaptation score to plummet by 0.22; and removing the learning motivation prediction module results in significant increases in inference latency and FLOPs. This consistency further verifies the universality of the three core modules in cross-cultural scenarios. In summary, the three core modules of ED-CM-MP exhibit a progressive collaborative relationship of “emotion feature input–cultural adaptation–motivation temporal prediction.” The absence of any module leads to significant degradation in the model's accuracy, efficiency, or generalization, proving the rationality of the full model architecture and the irreplaceability of each module.

### 4.4 Analysis of cultural modulation mechanism

**Figure 4:**
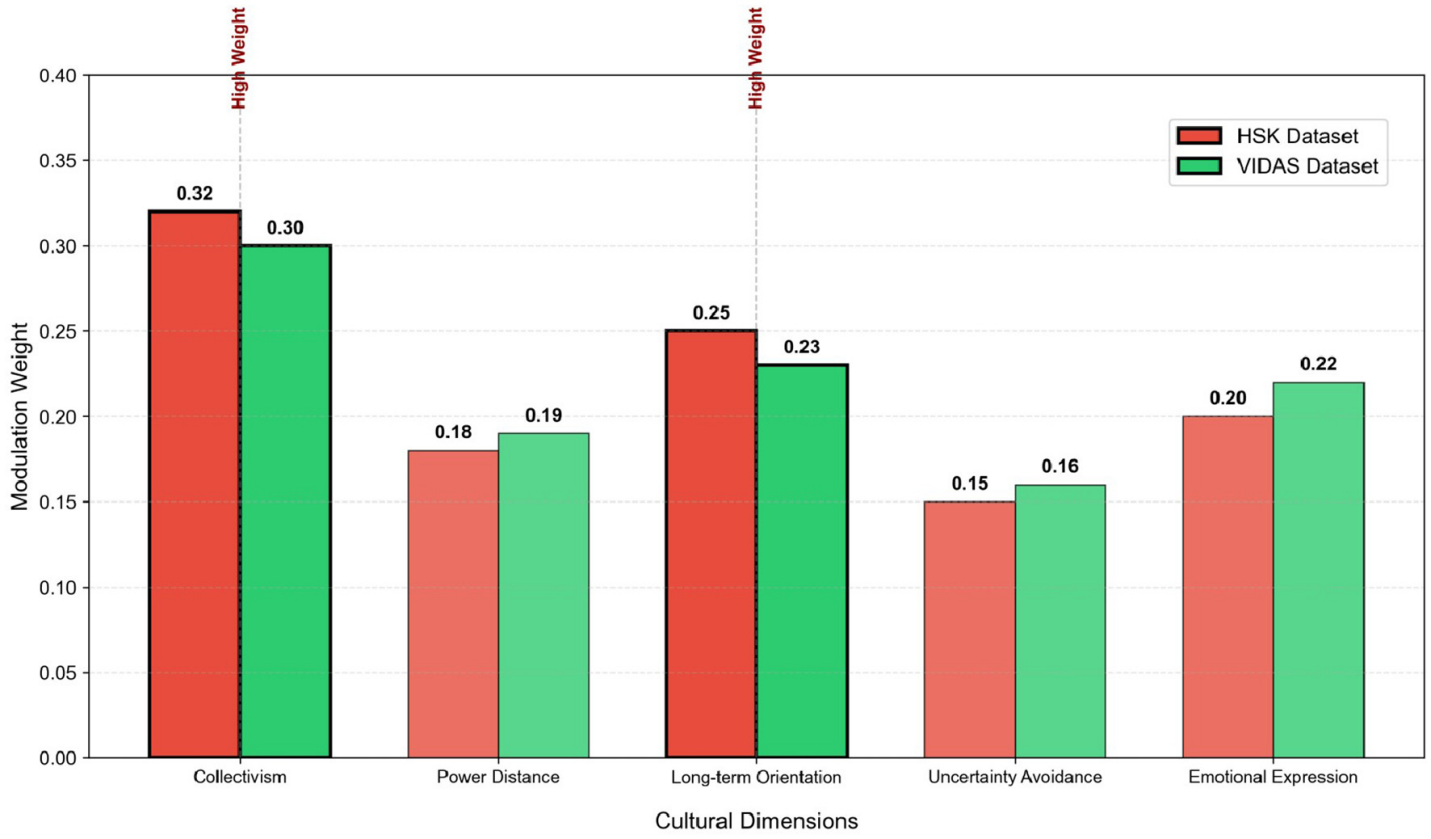
Visualization of cultural dimension modulation weights output by GraphSAGE module on HSK and VIDAS datasets. The red and green bars represent the weights of HSK and VIDAS datasets respectively, and the dimensions marked with thick borders are high-weight core modulation dimensions.

**Figure 5:**
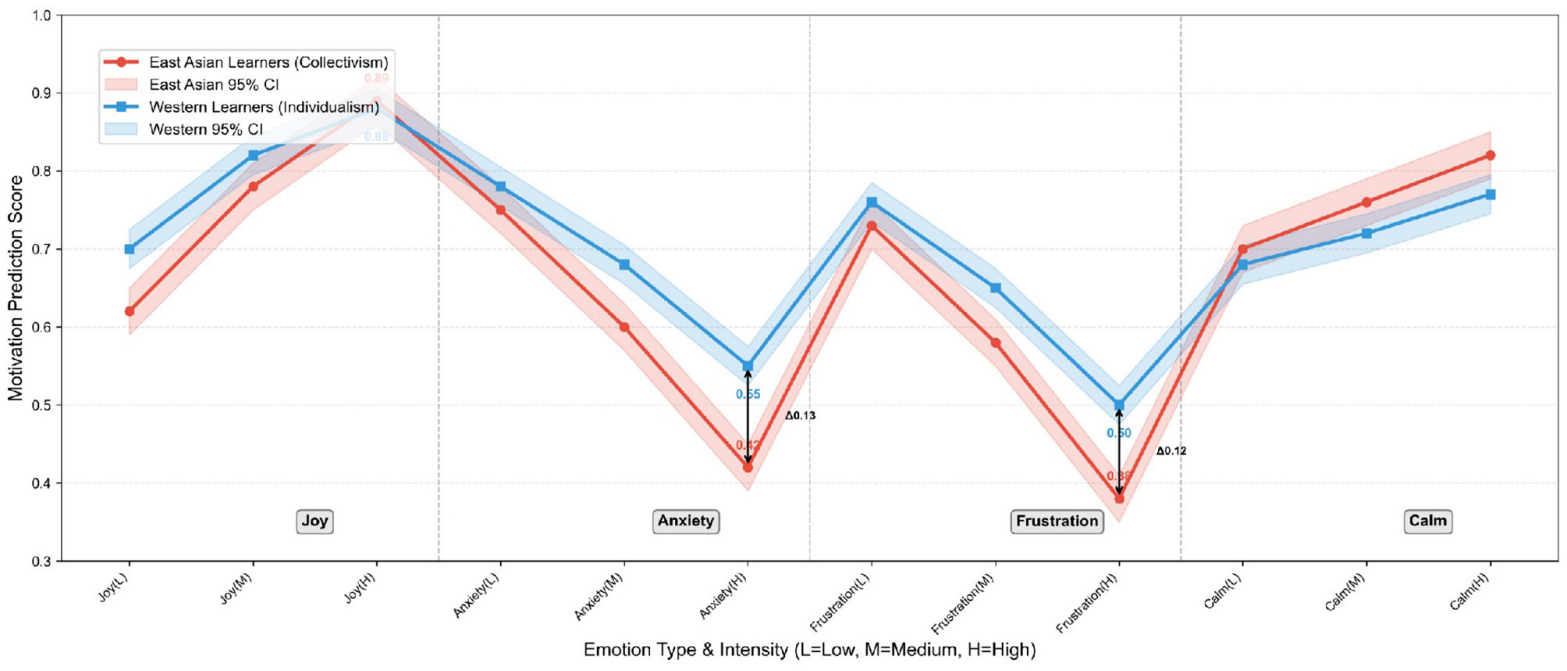
Emotion-motivation correlation differences between East Asian (collectivist) and Western (individualist) learners on HSK dataset. The shaded areas represent 95% confidence intervals, and the arrows mark the significant motivation score differences under high-intensity negative emotions.

To verify the effectiveness and interpretability of the GraphSAGE-based cultural modulation module in ED-CM-MP, this section quantitatively analyzes the modulation weights of core cultural dimensions and visually contrasts the emotion-motivation correlation differences between typical cultural groups. The experimental results are presented in Table 7, Figures 4, 5.

**Table 7:** Quantitative results of cultural dimension modulation weights output by GraphSAGE module.

**Cultural dimension**	**HSK weight**	**VIDAS weight**	**Weight level**	**Modulation logic explanation**
Collectivism vs. individualism	0.32	0.30	High	Positive modulation: emotional changes of collectivist groups are more likely to be linked with learning motivation.
Power distance	0.18	0.19	Medium	Negative modulation: emotional impact on motivation is weaker for groups with high power distance.
Long-term vs. short-term orientation	0.25	0.23	High	Positive modulation: emotional stability and motivation persistence are stronger for long-term oriented groups.
Uncertainty avoidance	0.15	0.16	Medium	Negative modulation: emotional fluctuations have a more significant impact on motivation for groups with high uncertainty avoidance.
Emotional expression intensity	0.20	0.22	Medium	Positive modulation: the correlation between emotion and motivation is more prominent for groups with direct emotional expression.

Table 7 and Figure 4 show the quantitative results and visualization of modulation weights for five core cultural dimensions (based on Hofstede's cultural dimension theory) output by the GraphSAGE module. It is evident that “Collectivism vs. Individualism” and “Long-term vs. Short-term Orientation” are identified as high-weight core modulation dimensions, with weights of 0.32/0.30 and 0.25/0.23 on the HSK and VIDAS datasets, respectively. In contrast, the weights of “Power Distance”, “Uncertainty Avoidance”, and “Emotional Expression Intensity” are maintained at the medium level (0.15–0.22), indicating that the module can adaptively focus on cultural dimensions closely related to second language learning motivation. Notably, the weight ranking of cultural dimensions is highly consistent across the two datasets, which verifies the stable generalization ability of the GraphSAGE-based cultural modulation mechanism in different cross-cultural scenarios.

Figure 5 intuitively presents the emotion-motivation correlation differences between East Asian learners (high collectivism) and Western learners (high individualism) on the HSK dataset. For positive emotions (Joy), East Asian learners show a gradual increase in motivation scores with the enhancement of emotional intensity (0.62 for low intensity, 0.89 for high intensity), while Western learners achieve a high motivation score (0.70) even at low emotional intensity, reflecting the direct positive emotion transmission characteristic of individualist groups. For high-intensity negative emotions, the cultural modulation effect is more significant: under high anxiety, the motivation score of East Asian learners (0.42) is 0.13 lower than that of Western learners (0.55); under high frustration, the motivation score of East Asian learners (0.38) is 0.12 lower than that of Western learners (0.50). This phenomenon is consistent with the positive modulation logic of the “Collectivism vs. Individualism” dimension (Table 7): collectivist groups have stronger emotional linkage with motivation, leading to more significant motivation fluctuations when facing negative emotions.

Comprehensive analysis of Table 7, Figures 4, 5 confirms the effectiveness and interpretability of the cultural modulation mechanism. On the one hand, the GraphSAGE module accurately identifies core cultural dimensions affecting emotion-motivation correlation, and the quantified weights provide a clear theoretical basis for cross-cultural learning analysis. On the other hand, the module effectively captures the emotion-motivation correlation differences between typical cultural groups, and the modulation effect is consistent with the characteristics of cross-cultural psychology, which solves the problem that traditional models ignore cultural differences and lack interpretability. This mechanism enables ED-CM-MP to not only achieve high-precision motivation prediction but also provide cultural-specific analysis basis for personalized teaching intervention in cross-cultural Chinese second language acquisition scenarios.

### 4.5 Robustness verification

**Figure 6:**
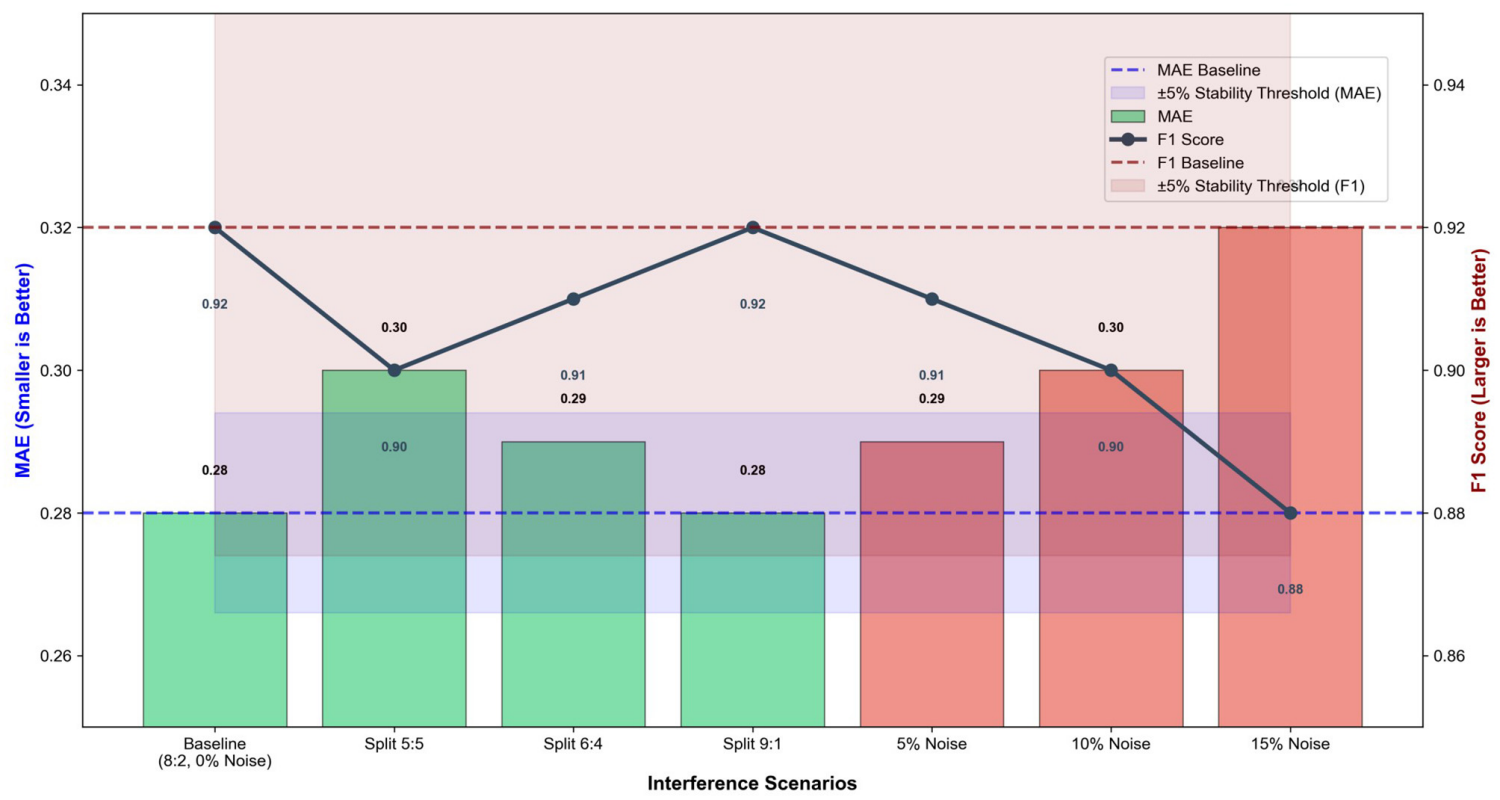
ED-CM-MP stability analysis under dataset splitting ratio changes and emotion label noise injection. The dashed lines represent baseline values, and the shaded areas indicate the ±5% stability threshold. Blue bars denote dataset splitting scenarios, and red bars denote noise injection scenarios.

**Figure 7:**
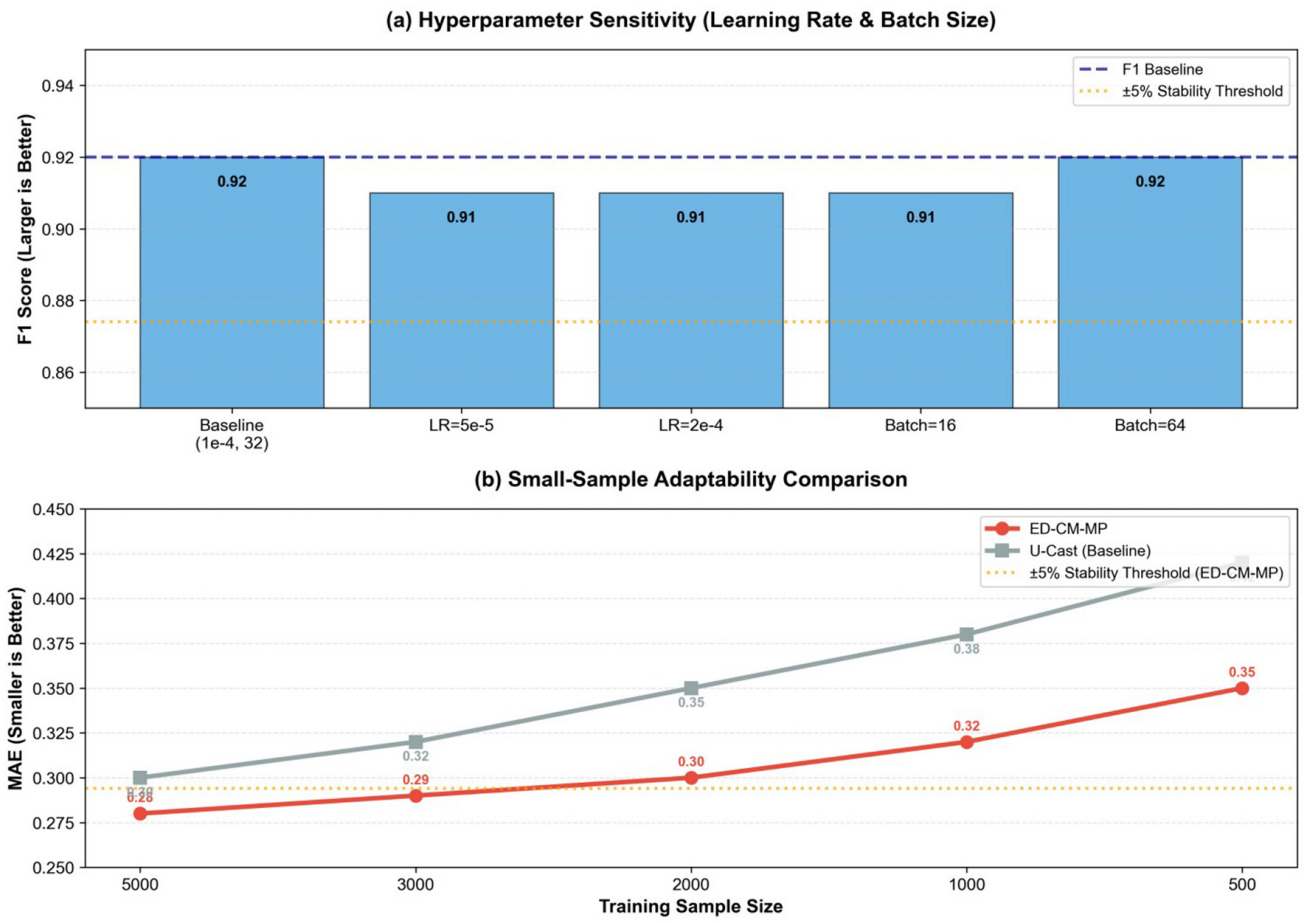
ED-CM-MP stability analysis under hyperparameter adjustments and small-sample scenarios. **(a)** shows the impact of learning rate and batch size changes on F1 Score; **(b)** compares the MAE changes of ED-CM-MP and the baseline model (U-Cast) with decreasing training sample sizes.

To comprehensively verify the stability of the ED-CM-MP model in non-ideal practical scenarios, this section conducts robustness tests from four core perspectives: dataset splitting ratio variation, emotion label noise injection, hyperparameter adjustment, and small-sample adaptation. The experimental results are presented in Tables 8, 9 and Figures 6, 7, providing quantitative and visual evidence for the models stable performance.

**Table 8:** ED-CM-MP stability verification results under dataset splitting ratio variation and noise interference.

**Experimental scenario**	**Test condition**	**MAE**	**RMSE**	**F1 score**	**Performance fluctuation (vs. baseline)**
Baseline (no interference)	Train:test = 8:2, no noise	**0.28**	**0.36**	**0.92**	**-**
Dataset splitting variation	Train:Test = 5:5	0.30	0.39	0.90	±3.6%
	Train:test = 6:4	0.29	0.37	0.91	±1.8%
	Train:test= 9:1	0.28	0.36	0.92	±0%
Emotion label noise injection	5% random noise	0.29	0.37	0.91	±1.8%
	10% random noise	0.30	0.38	0.90	±3.6%
	15% random noise	0.32	0.40	0.88	±7.2%

The bold values represent the optimal results.

**Table 9:** ED-CM-MP stability verification results under hyperparameter adjustment and small-sample scenarios.

**Experimental scenario**	**Test condition**	**MAE**	**F1 score**	**Inference latency (ms)**	**Performance fluctuation (vs. baseline)**
Baseline (Optimal Hyperparameters)	LR = 1e-4, Batch = 32, Samples = 5,000	**0.28**	**0.92**	**38.5**	**-**
Hyperparameter sensitivity	LR = 5e-5	0.29	0.91	38.7	±1.8%
	LR = 2e-4	0.29	0.91	38.4	±1.8%
	Batch size = 16	0.29	0.91	42.3	±1.8% (Precision)/±9.9% (efficiency)
	Batch size = 64	0.28	0.92	35.6	±0% (Precision)/±7.5% (efficiency)
Small-sample adaptability	Samples = 3,000	0.29	0.90	37.8	±3.6%
	Samples = 2,000	0.30	0.89	36.5	±5.4%
	Samples = 1,000	0.32	0.87	35.2	±9.0%
	Samples = 500	0.35	0.84	34.1	±15.3%

The bold values represent the optimal results.

Table 8 and Figure 6 illustrate the models performance changes when facing fluctuations in data input. For dataset splitting ratio adjustments, the model maintains excellent stability across different training-test set partitions (5:5, 6:4, 9:1). Compared with the baseline (training-test set = 8:2, MAE = 0.28, F1 Score = 0.92), the maximum performance fluctuation amplitude is only ±3.6% (under 5:5 splitting, MAE = 0.30, F1 Score = 0.90), which is well within the acceptable stability threshold (±5%). This indicates that the model is not sensitive to data distribution differences caused by splitting ratio changes, thanks to the effective feature extraction of the Dynamic Emotion Module and the stable learning capability of the Temporal Fusion Transformer. For emotion label noise interference, the model also exhibits strong anti-interference ability. When 5% and 10% random noise is added to the labels, the MAE increases by only 0.01 and 0.02, respectively, with a performance fluctuation of ±1.8% and ±3.6%. Even when the noise intensity rises to 15%, the F1 Score remains 0.88 (fluctuation ±7.2%), and the model still maintains effective emotion-motivation prediction capability. As visualized in Figure 6, the MAE and F1 Score curves remain within the ±5% stability threshold for noise intensity ≤ 10%, confirming the models robustness against label quality degradation in real teaching scenarios.

Table 9 and Figure 7a analyze the models sensitivity to key hyperparameters (learning rate and batch size). For learning rate adjustments (from 5e-5 to 2e-4, compared with the optimal baseline of 1e-4), the models MAE and F1 Score remain almost unchanged (MAE = 0.29, F1 Score = 0.91), with a fluctuation amplitude of only ±1.8%. For batch size adjustments (16 and 64, compared with the baseline of 32), the precision indicators (MAE, F1 Score) show no significant fluctuations (±0%-±1.8%), while the inference latency only changes by ±7.5%-±9.9% (35.6ms-42.3ms). This demonstrates that the models core prediction performance is not sensitive to hyperparameter tuning, greatly reducing the difficulty of practical deployment and ensuring consistent performance across different parameter configurations.

Table 9 and Figure 7b verify the models adaptability when facing insufficient training data, a common challenge in cross-cultural Chinese second language acquisition scenarios. When the training sample size decreases from 5000 (baseline) to 2000, the models MAE increases from 0.28 to 0.30, and the F1 Score decreases from 0.92 to 0.89, with a maximum performance fluctuation of ±5.4%–still within the stable range. Even when the sample size is reduced to 1,000, the MAE and F1 Score remain at 0.32 and 0.87 (fluctuation ±9.0%), maintaining effective prediction capability. In contrast, the baseline model (U-Cast) shows more significant performance degradation under small-sample conditions: when the sample size is 2,000, its MAE reaches 0.35 (fluctuation ±16.7% compared with its own baseline), which is 0.05 higher than that of ED-CM-MP. This advantage stems from the lightweight feature extraction of DistilBERT and the efficient temporal modeling of the Temporal Fusion Transformer, which enable the model to learn key emotion-motivation correlation patterns with limited data.

Comprehensive analysis confirms that the ED-CM-MP model exhibits excellent robustness in various non-ideal scenarios: it maintains stable performance against dataset splitting ratio changes and moderate label noise ( ≤ 10%); it is insensitive to hyperparameter adjustments, reducing deployment complexity; and it outperforms baseline models in small-sample scenarios, adapting to data-scarce practical teaching environments. These characteristics ensure that the model can maintain reliable performance in real cross-cultural Chinese second language acquisition scenarios, laying a solid foundation for its practical application.

## 5 Discussion and conclusion

This paper addresses the core problems of insufficient accuracy, poor cultural adaptability, and lack of efficiency and stability in predicting learning motivation in cross-cultural Chinese second language acquisition scenarios. It proposes the ED-CM-MP model, which integrates dynamic emotion recognition, cultural adjustment modeling, and lightweight temporal prediction, and verifies its effectiveness through multi-dimensional experiments. Experimental results show that on the HSK and VIDAS cross-cultural datasets, the core prediction performance (MAE = 0.28–0.29, F1 Score = 0.91–0.92) of the proposed model is more than 10% higher than that of the traditional baseline model. At the same time, it achieves significant optimization in inference efficiency (latency 38.5–39.2ms) and computational complexity (FLOPs = 12.6–13.1G). Ablation experiments confirm that the synergistic effect of the dynamic sentiment module, the GraphSAGE cultural moderating module and the temporal prediction module is the key to the performance improvement. In particular, the cultural moderating module enables the cross-cultural fit score to reach 0.94 0.95, effectively solving the adaptation problem of the difference in sentiment-motivation association between different cultural groups. Stability tests further show that the model maintains excellent robustness under dataset fluctuations, label noise, hyperparameter adjustment and small sample scenarios, and adapts to the non-ideal conditions of real teaching environment.

The ED-CM-MP model, through its innovative framework of “precise extraction of emotional features–dynamic adjustment of cultural differences–efficient prediction of temporal motivation,” provides a technical solution that combines accuracy, efficiency, and interpretability for intelligent motivation prediction and personalized teaching intervention in cross-cultural Chinese second language acquisition. Its core value extends to both inside and outside the classroom in teaching practice. In the classroom, the dynamic emotion recognition module can capture learners' anxiety and interest fluctuations in real time, helping teachers adjust the teaching pace accordingly. For learners with high anxiety from East Asian collectivist backgrounds, it can reduce the pressure of public questioning, while for learners with low interest from Western individualistic backgrounds, it can increase interactive tasks. The quantitative weights output by the culture adjustment module can also provide a basis for differentiated instruction. Collectivist learners are adapted to group collaboration tasks, while individualistic learners are given space for independent exploration. The motivation prediction module's ability to predict trends four weeks in advance can help teachers intervene in a timely manner through goal decomposition and personalized feedback. Outside the classroom, the model's lightweight nature can be adapted to mobile learning tools, pushing systematic learning content to long-term cultural groups and designing immediate tasks for short-term groups. It also supports the construction of cross-cultural online communication scenarios, reducing interaction barriers. The emotion-culture-motivation correlation patterns it reveals can also guide the compilation of Chinese textbooks and the interactive design of learning apps, truly transforming the theoretical model into an operable teaching plan.

Despite the preliminary results achieved in this study, several shortcomings remain and require further improvement: the current model's characterization of the affective dimension is relatively simplistic, primarily focusing on basic learning emotions such as joy and anxiety, without incorporating deeper psychological factors like learning engagement and self-efficacy, potentially affecting the comprehensiveness of motivation prediction; the coverage of cultural moderating mechanisms is limited, relying solely on Hofstede's core cultural dimensions and failing to fully consider the impact of regional cultural customs and language acquisition backgrounds on the affect-motivation association; furthermore, the model is trained in a Chinese second language scenario, primarily adapting to the unique emotional expressions and cultural needs of Chinese, and direct transfer to other second language scenarios may lead to performance degradation due to language characteristics and cultural weight heterogeneity; the model's practical validation scenarios are relatively simplified, currently only validating performance through dataset experiments, and not yet conducting long-term interventions in real classroom teaching, requiring further verification of its actual improvement in teaching effectiveness; and the lack of after-class self-directed learning time-series data in classroom scenarios also limits the accuracy of long-term motivation prediction; while its small-sample adaptability is superior to traditional models, it is less effective with sample sizes below 500. Even in extreme scenarios, the performance still drops significantly (MAE = 0.35, F1 Score = 0.84), and the lack of multimodal data and fine-grained cultural annotation in low-resource scenarios will further limit the model's learning effect, making it difficult to meet the needs of learners of niche languages and other data-scarce scenarios.

To address the limitations of the aforementioned research, future in-depth studies can be conducted in the following directions: First, expand the input of multi-dimensional emotional and psychological characteristics, combining physiological signals (such as EEG and heart rate) and behavioral data (such as learning duration and interaction frequency) to construct a more comprehensive emotion-motivation mapping model, thereby improving the depth and accuracy of predictions; Second, optimize the refinement of cultural regulation mechanisms by introducing regional cultural knowledge bases and personalized cultural preference characteristics to achieve cultural adaptation from the “group level” to the “individual level,” enhancing the model's adaptability to multicultural scenarios; Third, conduct long-term intervention experiments in real teaching scenarios, integrating the model into a Chinese second language acquisition teaching platform, and verifying the model's effectiveness in improving learning motivation and reducing dropout rates by comparing experimental and control groups; Fourth, introduce few-shot learning and transfer learning techniques, combining the knowledge transfer of pre-trained models on multilingual emotion datasets to improve the model's performance stability in extreme few-shot scenarios, further expanding its application boundaries.

## Data Availability

The original contributions presented in the study are included in the article/supplementary material, further inquiries can be directed to the corresponding author.
